# Reciprocal intraguild predation and predator coexistence

**DOI:** 10.1002/ece3.4211

**Published:** 2018-06-11

**Authors:** Renata Vieira Marques, Renato Almeida Sarmento, Adriana Gonçalves Oliveira, Diego de Macedo Rodrigues, Madelaine Venzon, Marçal Pedro‐Neto, Angelo Pallini, Arne Janssen

**Affiliations:** ^1^ Department of Entomology Federal University of Viçosa Viçosa Minas Gerais Brazil; ^2^ Federal University of Tocantins (UFT) Gurupi Tocantins Brazil; ^3^ Agriculture and Livestock Research Enterprise of Minas Gerais (EPAMIG) Viçosa Minas Gerais Brazil; ^4^ Department of Evolutionary and Population Biology IBED University of Amsterdam Amsterdam The Netherlands

**Keywords:** biological control, bistability, extinction, *Jatropha curcas*, population dynamics, predator‐prey interactions, stage structure

## Abstract

Intraguild predation is a mix of competition and predation and occurs when one species feeds on another species that uses similar resources. Theory predicts that intraguild predation hampers coexistence of species involved, but it is common in nature. It has been suggested that increasing habitat complexity and the presence of alternative food may promote coexistence. Reciprocal intraguild predation limits possibilities for coexistence even further. Habitat complexity and the presence of alternative food are believed to promote coexistence. We investigated this using two species of predatory mites, *Iphiseiodes zuluagai* and *Euseius concordis*, by assessing co‐occurrence in the field and on arenas differing in spatial structure in the laboratory. The predators co‐occured on the same plants in the field. In the laboratory, adults of the two mites fed on juveniles of the other species, both in the presence and the absence of a shared food source, showing that the two species are involved in reciprocal intraguild predation. Adults of *I. zuluagai* also attacked adults of *E. concordis*. This suggests limited possibilities for coexistence of the two species. Indeed, *E. concordis* invariably went extinct extremely rapidly on arenas without spatial structure with populations consisting of all stages of the two predators and with a shared resource. Coexistence was prolonged on host plant leaves with extra food sources, but *E. concordis* still went extinct. On small, intact plants, coexistence of the two species was much longer, and ended with the other species, *I. zuluagai*, often going extinct. These results suggest that spatial structure and the presence of alternative food increase the coexistence period of intraguild predators.

## 1 INTRODUCTION

Intraguild predation (IGP, hereafter) is a mix of competition and predation and occurs when one species feeds on another species that uses the same resources (Polis & Holt, 1992). In the last decades, IGP has received considerable attention because it commonly occurs in many ecosystems (Arim & Marquet, 2004; Holt & Polis, 1997; Morin, 1999; Polis & Holt, 1992; Polis, Myers, & Holt, 1989; Polis & Winemiller, 1996; Rosenheim, 2007; Rosenheim, Kaya, Ehler, Marois, & Jaffee, 1995). Theoretical models of intraguild predation usually consider three species: the shared resource, the IG‐prey, and the IG‐predator, and predicts that all three species can coexist only if the IG‐prey is the better competitor for the shared resource (Holt & Polis, 1997). Even then, however, the parameter space for coexistence is limited to intermediate levels of productivity (Diehl & Feissel, 2000; Holt & Polis, 1997; Mylius, Klumpers, de Roos, & Persson, 2001) hence, IGP is not predicted to be common. This discrepancy between theory and reality has resulted in a quest for factors that increase the probability of coexistence of species involved in intraguild predation, such as temporal variation, alternative prey, and spatial structure (Amarasekare, 2007; Daugherty, Harmon, & Briggs, 2007; Holt & Huxel, 2007; Janssen, Sabelis, Magalhães, Montserrat, & van der Hammen, 2007; Mylius et al., 2001; Rosenheim, 2007; Rudolf, 2007; Vance‐Chalcraft, Rosenheim, Vonesh, Osenberg, & Sih, 2007).

The stage or size of individuals often determine whether they are vulnerable or invulnerable to predation (Claessen, Van Oss, de Roos, & Persson, 2002; de Roos, Leonardson, Persson, & Mittelbach, 2002) and in the case of intraguild predation, whether they are predators or prey (Choh, Ignacio, Sabelis, & Janssen, 2012; Montserrat, Magalhaes, Sabelis, de Roos, & Janssen, 2012). In such size‐structured systems, two predator species can attack each other’s vulnerable stages (Choh et al., 2012; Montserrat et al., 2012; Polis, 1984), thus engaging in reciprocal intraguild predation. This interaction occurs in natural systems but has not received much attention (Montserrat et al., 2012; Polis et al., 1989; van der Hammen, de Roos, Sabelis, & Janssen, 2010; Wissinger, 1992; Woodward & Hildrew, 2002). Modeling studies and experiments have shown that reciprocal IGP leads to mutual exclusion or alternative stable states, in which either one or the other competitor persists alone with the resource, depending on the initial densities (a so‐called priority effect, HilleRisLambers & Dieckmann, 2003; Montserrat et al., 2012; Schellekens & van Kooten, 2012; van der Hammen et al., 2010).

Intraguild predation and reciprocal IGP is not only important in determining species coexistence, but also from an applied perspective. They occur frequently in biological control systems (Rosenheim et al., 1995), where, in theory, they would disrupt pest control. Although this disruption does not seem to occur very often, it does in some systems (Janssen et al., 2006; Rosenheim & Harmon, 2006). We therefore investigated the occurrence of IGP and reciprocal IGP between two predatory mite species that are considered for biological control of pests of the biodiesel plant *Jatropha curcas* L., on which both species occur (Marques et al., 2015; Sarmento et al., 2011).

Predatory mites are often used for biological control (Gould, 1977; Huffaker & Kennett, 1953; Janssen & Sabelis, 2015; Messelink, van Maanen, van Steenpaal, & Janssen, 2008; Nomikou, Janssen, Schraag, & Sabelis, 2001; Ramakers, 1980; van Lenteren, Bolckmans, Köhl, Ravensberg, & Urbaneja, 2018; Yaninek & Hanna, 2003). Intraguild predation and reciprocal IGP is common in phytoseiidaes (Ferreira, Cunha, Pallini, Sabelis, & Janssen, 2011; Guzmán, Sahún, & Montserrat, 2016; Hatherly, Bale, & Walters, 2005; Montserrat, Janssen, Magalhães, & Sabelis, 2006; Schausberger & Croft, 2000), and the use of multiple species of predatory mites can therefore disrupt biological control (Rosenheim & Harmon, 2006; Rosenheim et al., 1995). We therefore studied the interactions between two species of predatory mites: *Iphiseiodes zuluagai* Denmark & Muma and *Euseius concordis* Chant. These two phytoseiidaes attack the broad mite, *Polyphagotarsonemus latus* (Banks) (Acari: Tarsonemidae) and the spider mite *Tetranychus bastosi* Tuttle, Baker & Sales (Acari: Tetranychidae) in *Jatropha curcas* plantations in Brazil (Sarmento et al., 2011). We investigated whether the two predators are involved in IGP or reciprocal IGP. As explained above, if IGP would occur between the two predator species, theory predicts limited possibilities for coexistence, and no coexistence in the case of reciprocal IGP. In both cases, theory predicts that combining the two species would not result in better pest control (Janssen et al., 2006; Rosenheim & Harmon, 2006; Rosenheim et al., 1995).

We therefore first assessed the possible co‐occurrence of *I. zuluagai* and *E. concordis* on the same *J. curcas* plants in the field, showing that the two species do co‐occur. This would not be expected when they engage in reciprocal IGP, and would be less likely to occur in the case of simple IGP. Subsequently, we show that the two species do engage in reciprocal IGP, and that one species indeed rapidly excludes the other species on small arenas without spatial structure in the laboratory. Coexistence between the two species was considerably prolonged, however, on plant leaves and on intact plants, suggesting that increased spatial structure promoted coexistence, explaining how the two species can coexist in the field while engaged in reciprocal IGP.

## 2 MATERIAL AND METHODS

### 2.1 The experimental system

The experimental system consisted of the two predatory mite species *I. zuluagai* and *E. concordis* and pollen of *Ricinus communis* L. as a shared resource. The life cycle of phytoseiidae mites comprises five stages: egg, larva, protonymph, deutonymph, and adult. The two phytoseiidaes can develop and reproduce on pollen as well as on *P. latus* and *T. bastosi*, the two predominant pest species occurring on *J. curcas* (Sarmento et al., 2011). This crop is becoming increasingly popular in the biodiesel industry in North Brazil. It is adapted to arid, stony and low fertility soils and can grow under a wide range of precipitation conditions (Gübitz, Mittelbach, & Trabi, 1999; Openshaw, 2000). For sustainable production of biodiesel, the main pests of this crop should be controlled without pesticides. Both predatory mites studied here have been considered for biological control of *P. latus* and *T. bastosi* in this crop.

### 2.2 Co‐occurrence of *I. zuluagai* and *E. concordis* in the field

Because theory predicts that co‐occurrence of the two species would not be likely when they would be engaged in IGP, we first assessed the co‐occurrence of *I. zuluagai* and *E. concordis* on the same plants. Twelve surveys were carried out on 30 *J. curcas* plants located around the city of Gurupi, Tocantins State, Brazil (11^o^48′29″S, 48^o^56′39″ W, 280 m altitude), during three periods (03 – 27 March, 08 – 27 May and 18 September – 24 October 2009). During each survey, nine leaves were collected from each plant, three from each canopy stratum (bottom third, medium third and top third), located from the fourth to eighth fully expanded leaf from the branches. Leaves were checked with a stereomicroscope (Tecnival SQF‐F, Brazil) and mites were identified by Dr. Farid Faraj from the University of Amsterdam, The Netherlands, and Dr. Manoel Guedes of the Federal Rural University of Pernambuco, Brazil. Voucher species of mites were deposited in the collection of the Laboratory of Entomology at the Federal University of Tocantins. Because *I. zuluagai* was not present in the field during the last period, only the first two periods could be analyzed. Because predatory mites can only cover short distances when walking and mainly disperse passively on air currents (Johnson & Croft, 1981; Sabelis & Dicke, 1985), the presence of the species on a plant within each of the other two periods probably depended on its presence on this plant earlier during the same period (hence, observations per plant are probably not independent). We therefore first scored whether each of the two species had been present on a plant during this entire period. To further verify the observed co‐occurrence patterns, we subsequently treated samples within a period as independent and analyzed co‐occurrence data per sampling date. The predators were often found not to co‐occur with the prey, which were therefore excluded from the analysis. The probability of co‐occurrence of *I. zuluagai* and *E. concordis* on the same plants was calculated following Griffith, Veech, and Marsh (2016), assuming a hypergeometric distribution.

### 2.3 Plant material and mite rearing

Predatory mites used for experiments were collected from natural populations on *J. curcas* plants in Gurupi, Tocantins state, Brazil (11°45′47″S, 49°02′57″W). They were reared inside plastic boxes (11 × 11 cm) on flexible plastic disks (∅ = 6 cm) floating on distilled water in a climate room at 28°C, 65%–70% R.H. and a 12 hr L/12 hr D photoperiod. Small tent‐like structures consisting of a folded piece of plastic with small pieces of cotton wool under it were supplied on the arenas, serving as oviposition substrate. A small quantity of castor bean (*Ricinus communis*) pollen was supplied daily on the arenas as food for the predatory mites (McMurtry & Scriven, 1964). The *I. zuluagai* colonies were also supplied with honey diluted to 50% with distilled water. Pollen was collected from native castor bean plants in the city of Gurupi, following the method described by Gravena, Benetoli, Moreira, and Yamamoto (1994) and was conserved in glass recipients in a refrigerator (6°C). The mites were manipulated with a small brush under a magnifying glass. Populations of predatory mites were regularly transferred to new arenas to prevent the development of undesired microorganisms. Cohorts of predators of the same age were obtained by incubating adult females from the rearing units on a new arena with ample pollen, allowing them to oviposit for 24 hr, after which they were removed and the eggs were reared until reaching the desired stage or age.

### 2.4 Intraguild predation in the presence and absence of a shared resource

Intraguild predation was measured both in the presence and absence of the shared resource (pollen, c. 1.5 × 10^−4^ g per day). Plastic arenas (∅ = 7.5 cm) as described previously were used. To verify whether adults of the two species fed on the juveniles of the other species, one gravid adult female of either *I. zuluagai* or *E. concordis* (9 days old since egg stage) and 30 protonymphs of the other species, all from cohorts of similar aged individuals, were put on an arena, either without or with ample *R. communis* pollen. Adult females were not starved before the experiment. We used protonymphs and not larvae because larvae would develop into protonymphs during the experiment and the first protonymphs might cannibalize the remaining larvae. Because predation events are not very frequent in predatory mites, it was not feasible to directly observe predation. We therefore assessed the numbers of protonymphs consumed and the numbers of eggs produced by the adult female predators after 48 hr. Arenas with 30 protonymphs of *E. concordis* or *I. zuluagai* without adult female predator were used to measure the natural mortality, and arenas with only an adult female predator and pollen were used to assess oviposition in the absence of intraguild predation. The experiment was replicated 10 times. The effect of the presence of an intraguild predator and the presence of alternative food on the proportion of dead juveniles of each species was analyzed with a generalized linear model (GLM) with a quasi‐binomial error distribution (logit link) to correct for overdispersion, with the number of surviving and dead juveniles as dependent variable. The numbers of eggs produced by the adult female predators were analyzed with a GLM with a Poisson (log link) error distribution with the treatments (with or without pollen) as main factors. Contrasts among treatments were assessed using the multcomp package with a Tukey test (Hothorn, Bretz, & Westfall, 2008). All analyses were performed with the statistical software R (R Development Core Team, 2017).

Intraguild predation does not only involve killing individuals of the competing species, but also feeding on it (Polis et al., 1989), which can be observed directly, or can be inferred from increased survival, reproduction or development due to the feeding on the competitor (Fonseca et al., 2017). When this is not the case, the interaction should be classified as interspecific killing, an extreme form of interference competition (Fonseca et al., 2017). The previous experiment showed some indications that the IG‐predators benefitted from feeding on IG‐prey. To further confirm this, we measured oviposition rates of adults when feeding on potential intraguild prey (same numbers as above) or without food on experimental arenas as described above. Because the oviposition of the first day is affected by the diet of the previous day (Sabelis, 1990), we did not include it in the analysis. After 48 hr, the numbers of eggs produced by the adult female predators were assessed. The experiment was replicated 10 times. Differences in the numbers of eggs were analyzed as explained above.

### 2.5 Population dynamics on an artificial arena with a shared resource

We evaluated the dynamics of mixed populations of the two predators in the presence or absence of pollen. Experiments were carried out on circular plastic arenas (∅ = 7.5 cm) inside plastic boxes (11 × 11 cm) filled with water. Ten adult females (9 days old since egg stage) of each predator species were placed on separate arenas and supplied with ample pollen. After 6 days, all life stages were present, and the number of adults, juveniles and eggs was assessed for each species. Subsequently, all individuals of both species were transferred to a clean arena, where the two species could interact. Arenas were supplied with either ample pollen or kept without pollen. The numbers of adults, juveniles, and eggs of the two predators were assessed every 2 days until one of the species went extinct. The two species are easily recognized because *I. zuluagai* is dark, including its eggs, and all stages of *E. concordis* are light. In our cultures, each species persisted for longer than the duration of this experiment when supplied with pollen, and we therefore did not include a control in which the predator species were kept separately. Pollen was refreshed every day. The experiment was replicated 10 times.

Because one of the two species went extinct 4 days after the populations of the two species were joined (hence, on day ten), the data of this day were strongly zero‐inflated and were not included in the statistical analysis. We therefore analyzed differences in the number of mites on day eight with a GLM with a quasi‐Poisson error distribution (log link), with the initial numbers on day six (when the two species were joined), and pollen and species as factors. Contrasts among treatments were assessed with general linear hypothesis testing with a Tukey correction for multiple comparisons (package lsmean, Lenth, 2016).

### 2.6 Intraguild predation on adults

Because rapid extinction of *E. concordis* was observed in the previous experiment, we suspected that adult females of *I. zuluagai* killed adults of *E. concordis*. To verify this, we quantified killing of the adults of *E. concordis* by adults of *I. zuluagai* in the presence of shared resource (pollen). Plastic arenas (∅ = 7.5 cm) were used as described above. One adult female of *I. zuluagai* (9 days old since egg stage) and five adult females of *E. concordis* (9 days old since egg stage) were put on an arena with ample pollen. A treatment without *I. zuluagai* served as control. After 48 hr, the numbers of adults of *E. concordis* eaten by the adult female of *I. zuluagai* were assessed. The experiment was replicated 10 times. Predation was analyzed with a generalized linear model (GLM) as above.

### 2.7 Population dynamics on plants

Because we observed that *E. concordis* went extinct extremely rapidly in mixed populations of all stages of the two predators on artificial arenas and we found no evidence of exclusion on *J. curcas* plants in the field, we evaluated the dynamics of mixed populations of the two predators on 30‐day‐old *J. curcas* plants with four leaves. Populations were prepared on plastic arenas (∅ = 7.5 cm) inside a plastic tray filled with water as described above. Ten females (9 days old since egg stage) of each predatory mite were placed on separate arenas and supplied with ample pollen. After 6 days, all life stages were present, and the numbers of adults were quantified for each species. Subsequently, all individuals of both species were transferred to the plants, where the two species were allowed to interact. In the first treatment, both *E. concordis* and *I. zuluagai* were placed on the newest leaf on the apical part of the plant. In the second treatment, *E. concordis* were placed on the newest leaf and *I. zuluagai* on the leaf below, and in the third treatment, *I. zuluagai* were placed on the newest leaf and *E. concordis* on the leaf below. A control was added with only *I. zulugai* to verify whether it could persist on plants.

The plants were supplied with 1.5 × 10^−4^ g of *R*. *communis* pollen daily, weighed on a precision balance (Shimadzu, Kyoto‐Japan) and placed on the leaves with the predators with a fine brush. The numbers of adults were quantified every 2 days until one of the species went extinct. It was impossible to count juveniles and eggs without destructive sampling. Each treatment was replicated 10 times. The numbers of mites were log(x + 1) transformed and were analyzed with a linear mixed‐effects model with treatment (i.e., release schedule) and time as fixed factors and time within plant as random factor. Contrasts among treatments were assessed as above. Differences in time to extinction of the populations were tested with a Cox proportional hazards model (Therneau, 2013).

### 2.8 Population dynamics on leaves with pollen and honey

Here, we evaluated the dynamics of the two predators on a leaf with leaf hairs and trichomes and in the presence of pollen and honey. The leaves of *J. curcas* are palmately veined, cordate to truncate at the base. Together, the venation and the trichomes form structures that potentially reduce encounters between the predators (Ferreira et al., 2011). Honey was added because the experiments above showed that *I. zuluagai* did not persist on plants with a diet of pollen alone. Leaves (diameter c. 5 cm) were placed inside plastic boxes (11 × 11 cm) filled with water. Ten females (9 days old since egg stage) of each predator species were placed on separate arenas and supplied with a large quantity of pollen and honey. After 6 days, all life stages were present, and the number of adults, juveniles, and eggs was assessed for each species. Subsequently, all individuals of both species were transferred to a clean leaf, where the two species were allowed to interact. The leaves were supplied with 1.5 × 10^−4^ of *R*. *communis* pollen daily and 3.7 × 10^−2^ g of honey once per week. The number of eggs, larvae, nymphs and adults of the two predators was assessed every 2 days until one of the species went extinct. Populations of only *I. zulugai* or *E. concordis* kept on the same arenas served as control. The experiment was replicated 10 times. The total numbers of mites (log(x + 10) transformed) were analyzed with a linear mixed‐effects model with treatment (alone or together) and time as fixed factors and time within replicate as random factor.

## 3 RESULTS

### 3.1 Co‐occurrence of *I. zuluagai* and *E. concordis* in the field

*Euseius concordis* was found in 64.6% of the samples and *I. zuluagai* occurred in 21.7% of the samples. Analysis of the overall co‐occurrence data of the two entire sampling periods in which both species occurred showed that the observed values were not significantly different from expected. Hence, there was no evidence that the two species excluded or avoided each other (Table 1). The number of plants on which the two species co‐occurred was significantly higher than expected from an independent distribution in three of the eight sampling dates, and it was not significantly higher on the other 5 days (Table 1). We conclude that the two species do co‐occur at least during several generations on the same plants in nature. Because theory predicts that possibilities for co‐occurrence are reduced when species are engaged in IGP, we subsequently tested the occurrence of IGP and reciprocal IGP in the laboratory.

**Table 1:** Co‐occurrence of the predatory mites *E. concordis* and *I. zuluagai* on *Jatropha curcas* plants in the field

Period^a^	*E. concordis* b	*I. zuluagai* b	Both	Exp(both)^c^	*p* d
March overall	28	6	6	5.6	0.634
3	9	3	3	0.9	**0.02**
10	25	3	2	3.5	0.43
18	17	4	3	2.3	0.41
27	10	0	0	0	
May overall	29	22	20	21.3	1
8	25	13	13	10.8	**0.043**
15	22	9	9	6.6	**0.035**
22	28	12	12	11.2	0.35
29	19	8	5	5.1	0.64

Note

^a^Sampling was performed in three periods, March, May, and September‐October 2009. Data from the last period are excluded because *I. zuluagai* was not encountered. Given are first overall co‐occurrence data (i.e., the presence/absence on plants during the entire month of March or May) and subsequently data for the four samples during the months with number of day (i.e., March 3rd, 10th); ^b^number of plants of 30 sampled on which a species was encountered, including plants on which the other species was also encountered; ^c^expected number of plants with co‐occurrence; ^d^probability of encountering both species the observed number of cases or more extreme, based on a hypergeometric distribution (Griffith et al., 2016). Significant values are given in bold.

### 3.2 Intraguild predation in the presence of a shared resource

**Figure 1:**
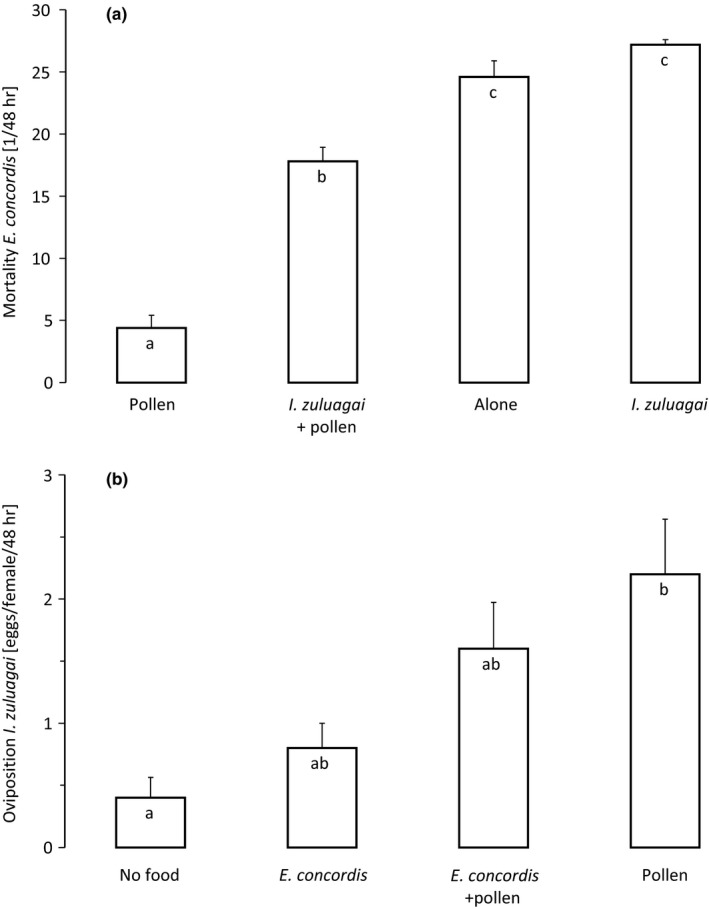
(a) Numbers of dead juvenile *E. concordis* (mean ± *SE*) after 48 hr in the presence or absence of ample pollen and in the presence or absence of adult *I. zuluagai*. (b) Oviposition rates (mean ± *SE*) after 48 hr of the adult *I. zuluagai* in the same experiments. Letters inside the bars indicate significant difference among treatments (contrasts after GLM)

The mortality of juveniles of *E. concordis* varied significantly among treatments (Figure 1a, GLM, *F*_3,36_ = 70.9, *p *<* *0.001). In the presence of large amounts of pollen, the mortality of protonymphs was higher in the presence than in the absence of adult females of *I. zuluagai* (Figure 1a, 1st and 2nd bar). The mortality of juvenile *E. concordis* was higher without pollen than with pollen (Figure 1a, 2st and 4th bar). The mortality did not differ between the two treatments without pollen, probably because it was very high in both cases (Figure 1a, 3rd and 4th bar). Oviposition by *I. zuluagai* was significantly affected by treatment (Figure 1b, GLM, *χ* ^*2*^ = 16.5, *df* = 3, *p* = 0.0009), and it was more than twice higher in the presence of pollen than in its absence (Figure 1b, compare first two bars with last two bars). Feeding on juveniles of *E. concordis* resulted in an increase in oviposition, but this effect was not significant (Figure 1b, compare 1st with 2nd bar and 3rd with 4th).

**Figure 2:**
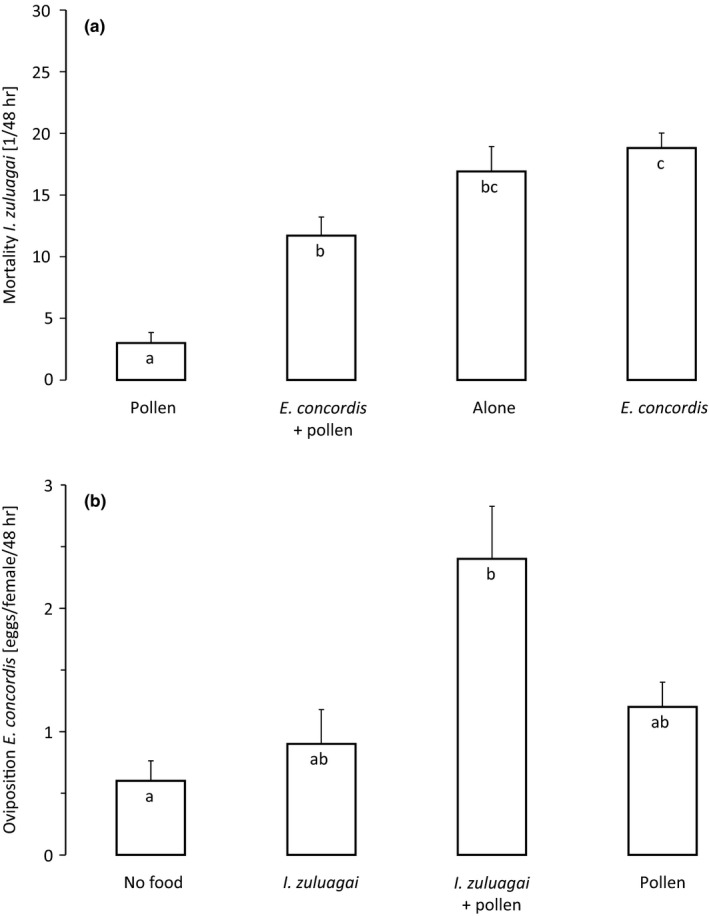
(a) Numbers of dead juvenile *I. zuluagai* (mean ± *SE*) after 48 hr in the presence or absence of ample pollen and in the presence or absence of one adult female *E. concordis*. (b) Oviposition rates (mean ± *SE*) after 48 hr of the adult *E. concordis* in the same experiments. Letters inside the bars indicate significant difference among treatments (contrasts after GLM)

The mortality rate of juvenile *I. zuluagai* also differed significantly among treatments (Figure 2a, GLM, *F*_3,36_ = 22.4, *p *<* *0.001). In the presence of pollen, the mortality of juvenile *I. zuluagai* was significantly higher in the presence of adult female *E. concordis* than in its absence (Figure 2a, cf. 1st with 2nd bar). Mortality of juvenile *I. zuluagai* was significantly higher without pollen than with it (Figure 2a, cf. the 1st with the 3rd bar and the 2nd with the 4th bar). The mortality did not differ between the two treatments without pollen, but was not very high. Perhaps the adult *E. concordis* fed mainly on starving and dying protonymphs, which may be easier to capture. The type of diet affected the oviposition rate of *E. concordis* (Figure 2b, GLM, *χ* ^*2*^ = 13.6, *df* = 3, *p* = 0.003). The addition of protonymphs of *I. zuluagai* increased the oviposition rate of *E. concordis*, but this effect was not significant (Figure 2b, cf. the 1st with 2nd bar and 3rd with 4th bar). The oviposition rate of *E. concordis* was highest when the mites were feeding on pollen and protonymphs of *I. zuluagai* (Figure 2b).

**Figure 3:**
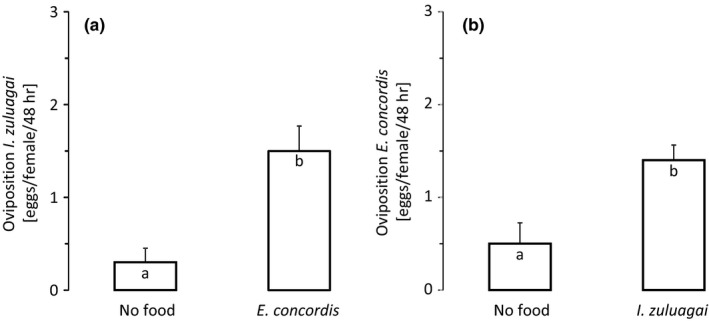
(a) Numbers of eggs (mean ± *SE*) produced by (a) adult female predatory mites *I. zuluagai* when feeding on juvenile *E. concordis*, and by (b) adult female predatory mites *E. concordis* when feeding on juvenile *I. zulugai*. Adult females of both species were allowed to feed on the juveniles of the other species for 2 days, but only oviposition rates of the second day were included to avoid effects of previous diet. Different letters inside the bars indicate significant differences between treatments

Because of the nonsignificant trends of an increase in oviposition by the IG‐predators in the presence of IG‐prey, we specifically tested oviposition in a separate experiment. The oviposition by *I. zuluagai* was significantly higher in the presence of juveniles of *E. concordis* than without food (Figure 3a, GLM, *df* = 1, 38, *χ* ^*2*^ = 9.0, *df* = 1, *p* = 0.003). Likewise, the oviposition by *E. concordis* was also significantly higher in the presence of juvenile *I. zuluagai* than without food (Figure 3b, GLM, *χ* ^*2*^ = 4.3, *df* = 1, *p* = 0.04). Together, this shows that the two species are involved in reciprocal intraguild predation (Fonseca et al., 2017).

### 3.3 Population dynamics on a shared resource

**Figure 4:**
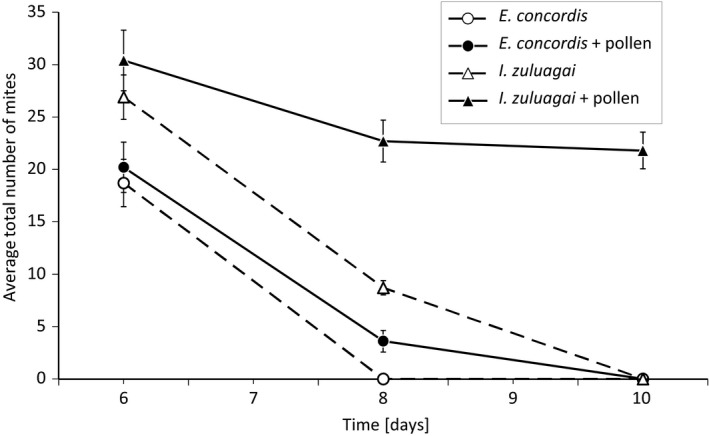
Mean total (all stages) numbers of mites (mean ± *SE*) of *I. zuluagai* (triangles) and *E. concordis* (circles). Populations of both species were started with 10 adult females and were allowed to grow on pollen for 6 days. Subsequently, populations of the two species were released on the same arena either with (drawn lines) or without (interrupted lines) pollen as a shared food source. See text for further explanation

The populations of both species increased in the period preceding the mixing of the populations from 10 individuals to 20–30 individuals on the 6th day, confirming our experience with rearing both species on a diet of pollen. After joining the two populations of predators on day 6, *E. concordis* invariably went extinct within 4 days, even in the presence of pollen (Figure 4). The population of *I. zuluagai* persisted only in the presence of pollen. The densities of both species on day 8 was significantly affected by the numbers of predators present when the two species were joined (GLM, *F*_1,36_ = 5.32, *p* = 0.027) and by the interaction between the presence of pollen and the other species (*F*_1,35_ = 419.2, *p* = 0.0001). This interaction was caused by the densities of *I. zuluagai* being affected by the presence of pollen, but those of *E. concordis* not (Figure 4, general linear hypothesis testing after GLM).

The rapid extinction of the populations of *E. concordis* suggests that *I. zuluagai* did not only target juveniles and perhaps eggs of the other species, but also adults. Indeed, the number of adult *E. concordis* decreased from 18.7 ± 2.26 (mean ± *SE*) to zero in the absence of pollen, and from 20.2 ± 2.39 to 3.6 ± 1.03 individuals in the presence of pollen. As intraguild predation on adult females is rare in Phytoseiidae, we further confirmed this in the following experiment.

### 3.4 Intraguild predation on adults

The mortality of adult *E. concordis* was much higher in the presence of an adult *I. zuluagai* than in its absence (average mortality with *I. zuluagai* ± *SE*: 0.42 ± 0.07; without: 0.04 ± 0.027; GLM, *χ* ^*2*^ = 23.0, *df* = 1, *p *<* *0.0001). There was no mortality of adult *I. zuluagai*.

### 3.5 Population dynamics on plants

**Figure 5:**
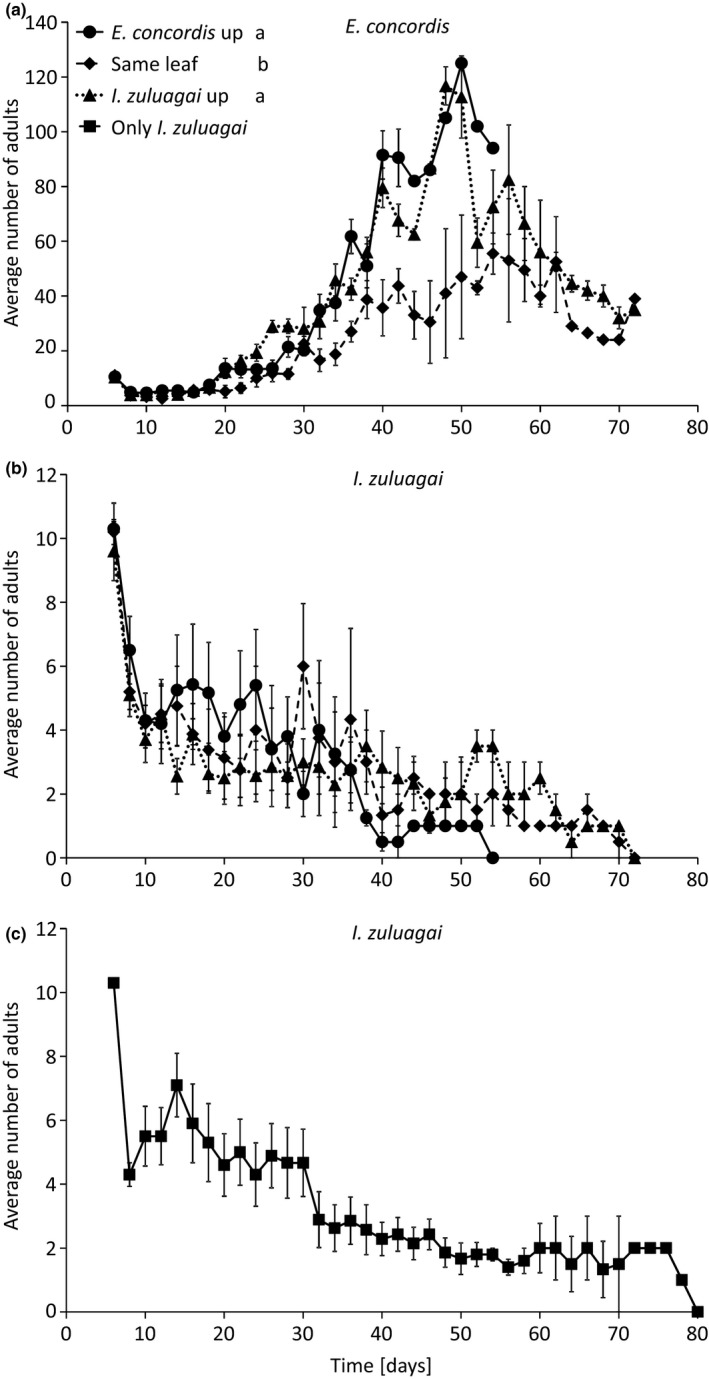
Mean ± *SE* numbers of adults of *E. concordis* (a) and *I. zuluagai* (b) per plant. Populations of both species started with 10 adult females and were allowed to grow on pollen for 6 days. Both species were either released on the upper leaf (Same leaf, diamonds) or one of the species was released on the upper leaf and the other one leaf down (*E. concordis* up and *I. zuluagai* up). (C) Only individuals of *I. zuluagai* were released. See text for further explanation. The letters in the legend show significance of differences among treatments for *E. concordis* (contrasts after lme). Treatments did not differ significantly for *I. zuluagai*

In contrast to the experiments on artificial arenas, the two populations now persisted for a much longer period (Figure 5). There was a significant effect of the interaction between treatment and time on the average densities of *E. concordis* (LME, *χ* ^*2*^ = 10.4, *df* = 2, *p* = 0.0054): densities were significantly lower when it was released on the same leaf as *I. zuluagai* (Figure 5a). Two and one populations of *E. concordis* went extinct before *I. zuluagai* when both species were released on the same leaf or when *I. zuluagai* was released on the highest leaf, respectively. In all other replicates of all treatments, *I. zuluagai* went extinct first. There was no significant difference in densities of *I. zuluagai* among treatments (Figure 5b, LME, *χ* ^*2*^ = 4.17, *df* = 2, *p* = 0.12). Time to extinction of populations of *I. zuluagai* did not differ significantly among treatments (Cox proportional hazards, Likelihood ratio test = 3.56, *df* = 2, *p* = 0.17). This species eventually also went extinct when present alone on a plant (Figure 5c), and although it took somewhat longer to go extinct than in the presence of *E. concordis*, this difference was not significant (Cox proportional hazards, Likelihood ratio test = 3.17, *df* = 1, *p* = 0.075).

### 3.6 Population dynamics on leaves with pollen and honey

**Figure 6:**
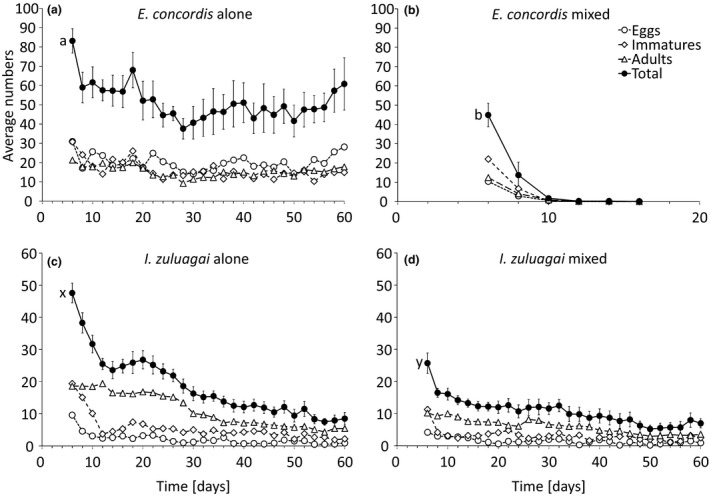
Mean numbers of eggs (open circles), immatures (open diamonds), adults (open triangles), and total mites (closed circles) of *E. concordis* (a and b) and *I. zuluagai* (c and d) per leaf, either alone (a, c) or together with the other species (b, d). Populations of both species started with 10 adult females and were allowed to grow on pollen and honey for 6 days. Subsequently, populations of the two species were released on the same leaf or on different leaves, in all cases with pollen and honey as food. Letters to the left of the total curves indicate a significant difference between the total numbers in the mixed and single populations. For reasons of clarity, *SE* are only shown for total numbers

The populations of both predator species alone persisted on a plant leaf with pollen and honey for the entire experimental period (Figure 6a and c). After joining the two predator populations on day 6, *E. concordis* invariably went extinct before day 16, whereas *I. zuluagai* persisted (Figure 6b and d). All stages went extinct at the same time, confirming that *I. zuluagai* attacked all stages of *E. concordis* (Figure 6b). For each species, there was a significant effect of the interaction between time and treatment (with or without the other species) on the total numbers of mites (LME, *E. concordis*:* χ* ^*2*^ = 19.7, *df* = 1, *p *<* *0.0001; *I. zuluagai*:* χ* ^*2*^ = 5.85, *df* = 1, *p* = 0.016). Contrasts between the two time series showed significant differences between treatments for both species (Figure 6). For *I. zuluagai*, the two time series differed in the first part of the experiment, but this difference disappeared in the second half (Figure 6c and d).

## 4 DISCUSSION

We show that the two species of predatory mites engaged in reciprocal intraguild predation, with adults of the two species feeding on each other’s juveniles. The adult females of both species showed a significant increase in reproduction on a diet consisting of juveniles of the other species (Figure 3), suggesting that they did not just kill the juveniles, but also fed on them. Furthermore, the adult females of *I. zuluagai* killed adults of *E. concordis*, causing *E. concordis* going extinct extremely rapidly in mixed populations of all stages of the two predators on arenas without spatial structure (Figure 4). We do not know whether the adults of *I. zuluagai* benefited from feeding on adults of the other species, that is, whether this qualified as intraguild predation. Without pollen, we encountered no eggs of *I. zuluagai* at the end of the experiment, suggesting that the population of *I. zuluagai* was also going extinct, and could perhaps not reproduce on a diet of adult *E. concordis* only. Possibly, adult *I. zuluagai* killed the adults of the other species to avoid that these would kill the offspring of the adult female *I. zuluagai*.

Intraguild predation is a common phenomenon in nature (Arim & Marquet, 2004; Polis et al., 1989). In general, the IG‐predator feeds on the smaller stages of the IG‐prey, and it is less common that adults of one species attack the adults of the other species (Polis et al., 1989). Adults of *I. zuluagai* (dorsal shield on average 343 × 278 μm) are bigger than adult of *E. concordis* (309 × 207 μm) (Lofego, 1998) and this might render adults of *E. concordis* vulnerable to predation by adults of the former species.

On plants, the period of coexistence of both populations was much longer than on artificial arenas and on leaves. This was perhaps caused by the increased spatial structure offered by plants compared to artificial arenas. It is known that plant structures may reduce the encounter and predation rates between predators (Ferreira et al., 2011; Pozzebon, Loeb, & Duso, 2015; Roda, Nyrop, Dicke, & English‐Loeb, 2000; Schmidt, 2014). Habitat structure can reduce the effects of intraguild predation by reducing the strength of the interaction between intraguild predator and intraguild prey (Janssen et al., 2007). Probably, leaf structures such as trichomes may have affected the coexistence of the predators in our experiments. In contrast to the experiments on artificial arenas, *I. zuluagai* ultimately went extinct on the plants, and not *E. concordis*. Although the densities of *E. concordis* were significantly lower when it was released on the same leaf as *I. zuluagai* (Figure 5a)*, E. concordis* persisted and *I. zuluagai* went extinct. However, we found that *I. zuluagai* did not persist on plants with pollen only (Figure 5c) and the time to extinction was not significantly affected by the presence of *E. concordis* (Figure 5b,c). Perhaps the extinction of *I. zuluagai* was not caused by the interaction with *E. concordis*, but by the lack of a suitable diet. Indeed, when providing honey as a diet supplement on arenas consisting of single leaves, populations of *I. zuluagai* persisted, resulting in *E. concordis* again going extinct (Figure 6), in line with theoretical predictions of the effects of alternative food for intraguild predators (Daugherty et al., 2007; Holt & Huxel, 2007). Together, these results show that an interplay between spatial structure and alternative food sources determine coexistence and exclusion of species involved in intraguild predation (Pozzebon et al., 2015). This may explain the co‐occurrence of the two species on plants in the field. Possibly, the absence of *I. zuluagai* from the field in September – October, which is the end of the dry season in the study area (Cruz, Sarmento, Teodoro, Neto, & Ignacio, 2013), even from plants without *E. concordis* was also caused by the lack of a suitable diet.

In theory, reciprocal intraguild predation, as was found here, reduces possibilities for coexistence even more than simple IGP (HilleRisLambers & Dieckmann, 2003). Earlier experimental work on systems with reciprocal intraguild predation, but with adults being invulnerable to attacks by the other species, has shown the possibility of bistability in the dynamics (Montserrat, Magalhaes, Sabelis, de Roos, & Janssen, 2008; Montserrat et al., 2012). The mechanism behind this is that resident populations with a high density of adults can prevent the invasion of the other species by killing all its offspring. In the system studied here, this bistability is likely to occur less, because the adults of *E. concordis* are vulnerable to intraguild predation. Hence, *I. zuluagai* could probably even invade in populations with high densities of *E. concordis* adults. Hence, our experiments on plastic arenas suggest low probabilities for coexistence of these two predators in the field. In contrast, we found no evidence of exclusion of either of the species on *Jatropha* plants in the field, and prolonged periods of co‐occurrence on single plants in the laboratory. We suggest that plants offer structural complexity that reduces the strength of intraguild predation, thus resulting in increased coexistence of the two species.

## CONFLICT OF INTEREST

None declared

## AUTHOR CONTRIBUTIONS

RVM, RAS, AP, and AJ conceived the ideas and designed the methodology; RVM, AGO, and DMR collected the data; RVM, AJ, and RAS analyzed the data; RVM, RAS, AJ, AP, MV, and MPN led the writing of the manuscript. All authors contributed critically to the drafts and gave final approval for publication.

## DATA ACCESSIBILITY

Data are available on Dryad (https://doi.org/10.5061/dryad.m1v7416).
